# Morning glory syndrome with retinal detachment and literature review—a case report

**DOI:** 10.3389/fmed.2026.1819842

**Published:** 2026-06-16

**Authors:** Yanhong Ding, Ai Zhang

**Affiliations:** 1 Department of Ophthalmology, Gongshu District Wenhui Community Health Service Center (Gongshu District Hospital of Traditional Chinese Medicine), Hangzhou, China; 2 Department of Ophthalmology, Wenzhou TCM Hospital of Zhejiang Chinese Medical University, Wenzhou, China

**Keywords:** MGS, morning glory syndrome, optic disc dysplasia, optic neuropathy, retinal detachment

## Abstract

Morning glory syndrome (MGS) is a congenital abnormal optic disc eye disease. In 1970, Kindler first described it and reported the disease in detail. Its fundus performance resembled a blooming morning glory, so it was named. This disease is relatively rare, and it was first reported in 1985 in China.

## 1 Introduction

Morning glory syndrome (MGS) is a rare congenital disorder of optic disc dysplasia (1) and is infrequently encountered in clinical practice. Its main features include optic nerve defects, distinct retinal vascular abnormalities, glial proliferation and transformation, and peripapillary pigmentary changes. The condition is named for its fundus morphology resembling a blooming morning glory and is typically monocular. This paper reports a case of MGS with retinal detachment and reviews the literature regarding its definition, pathogenesis, pathology, epidemiology, diagnosis, differential diagnosis, complications, and treatment.

## 2 Case report

A 22-year-old woman presented to our ophthalmology clinic for a “driver’s physical examination disqualification.” After a comprehensive examination, no systemic abnormalities were found. The patient was born full-term via natural delivery to non-consanguineous parents, with no history of maternal pregnancy complications or specific medication use. Family history was unremarkable, and there were no systemic genetic abnormalities. There was no history of ocular or systemic trauma, surgery, or medication use.

**Figure 1:**
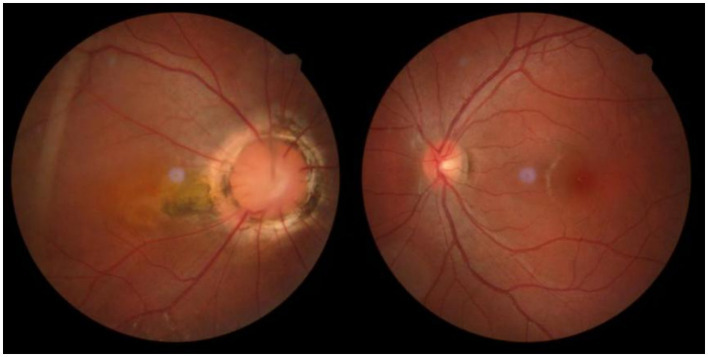
Colour photos of both eyes. After mydriasis, the optic disc of the right eye was enlarged by approximately 3–5 times compared with normal, pink, with a funnel-shaped deep depression in the centre. There were dense white villous tissues such as stamen filling in the centre. There was a grey-white choroidal retinal pigment ring in the depression around the optic disc. It was difficult to distinguish the central retinal artery and vein. There were more branches around the optic disc, about 20–26 branches, which were distributed from the optic disc depression to the surrounding retina. The pigment in the macular area was disordered, uplifted, and the fovea was not seen. The nasal measurement showed 1PD greyish brown deposition. There was no abnormality on fundus examination of the left eye.

**Figure 2:**
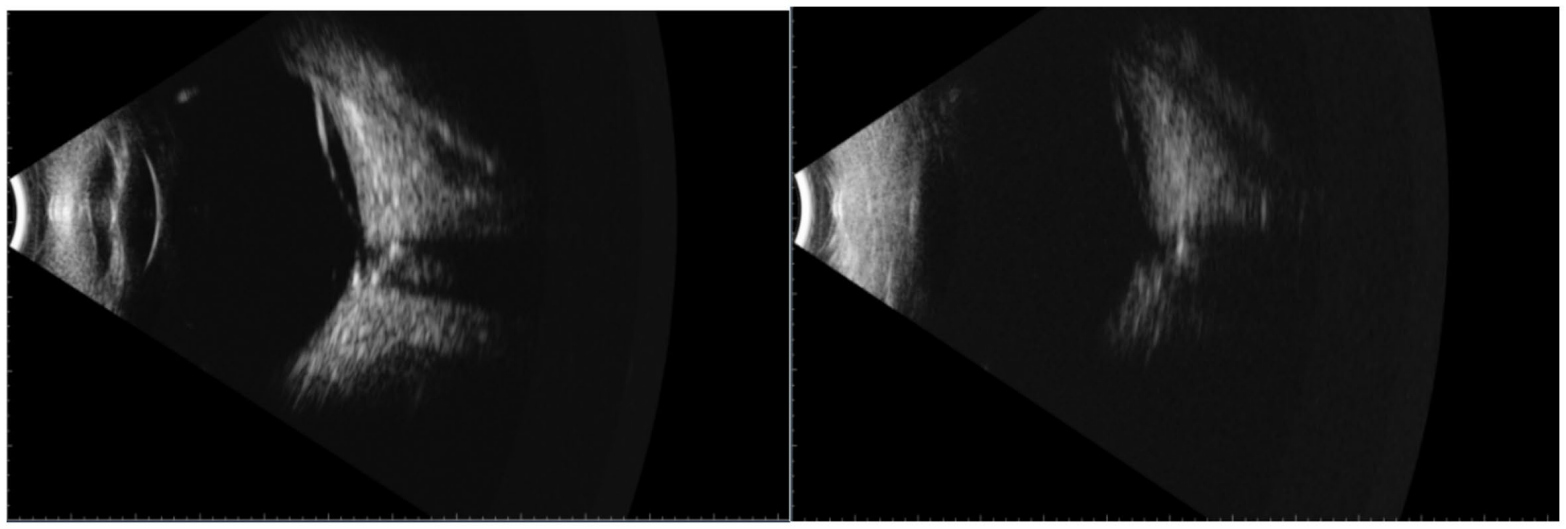
Ocular B-scan ultrasound (ACCUTOME A Halma company diagnostic ultrasound, USA, probe frequency 15 MHz) indicated: In the right eye, an arched white reflective band (retina) was seen floating within the vitreous cavity. Behind this band was an echo-free dark area. One end of the band connected to the optic disc. The optic disc region showed a localised, well-defined, regularly shaped depression communicating with the vitreous cavity. Its deep aspect extended posteriorly toward the optic nerve axis, forming a funnel-shaped dark area in the posterior pole. The lesion exhibited a slightly inverted “bottleneck” configuration. The optic disc appeared slightly enlarged and depressed, with irregular hypoechoic signals within the depression.

**Figure 3:**
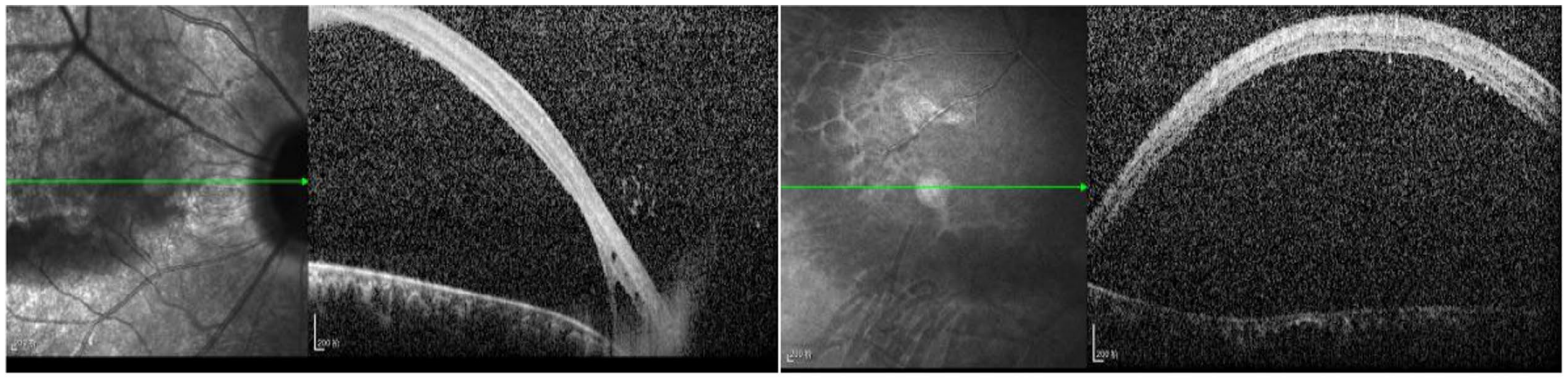
Optical coherence tomography (OCT) (Heidelberg Spectralis OCT, Germany) examination revealed separation between the retinal photoreceptor layer and the retinal pigment epithelium in the right eye, indicating retinal detachment.

**Figure 4:**
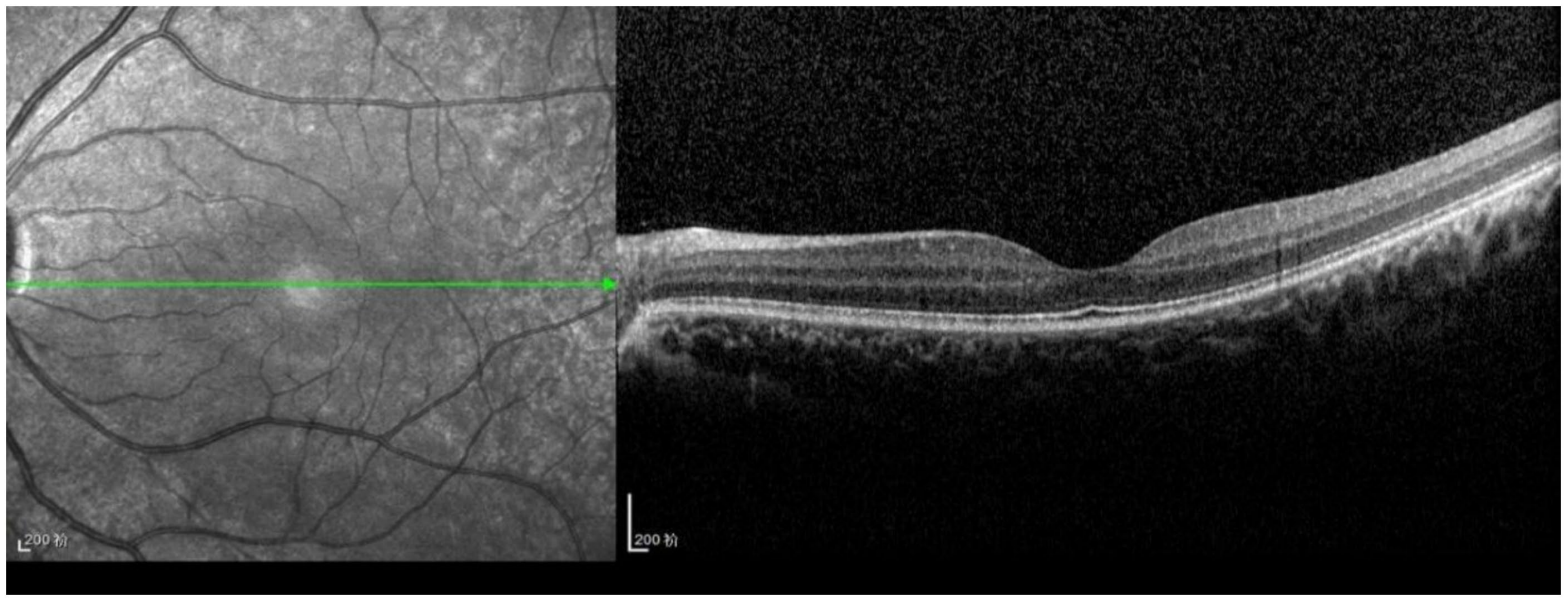
OCT examination of the left eye showed a normal macular structure. T is the most sensitive area of vision with a diameter of about 5 mm in the centre of the retina. Its 10 layers of structure are inner limiting membrane, nerve fibre layer, ganglion cell layer, inner plexiform layer, inner nuclear layer, outer plexiform layer, outer nuclear layer, outer membrane, rod-cone layer, and retinal pigment epithelium layer. These structures cooperate to complete the optical signal reception, processing, and transmission.

Specialist examination: Visual acuity (VA) was 0.01 in the right eye (*oculus dexter* [OD]) and 1.2 in the left eye (*oculus sinister* [OS]). Manifest refraction: OD: −0.50 = 0.01; OS: PL = 1.2. Intraocular pressure: right eye 13.8 mm Hg, left eye 15.2 mm Hg. The external eye showed no deformity, eye position was orthotropic, and anterior segment examination was unremarkable. Fundus examination after pupillary dilation (Nidec AFC-330, Japan) revealed, compared with the normal left eye, an enlarged optic disc in the right eye with a grey-white choroidal retinal pigment ring in the peripapillary depression. Numerous branching vessels were observed around the optic disc, and macular pigmentation was disorganised without a visible foveal reflex (Figure 1). Ophthalmic B-ultrasound (ACCUTOME A Halma, USA; probe frequency 15 MHz) demonstrated a suspended arc-shaped white retinal band within the vitreous cavity of the right eye, with an anechoic dark area behind the band. One end of the band was attached to the optic papilla, which appeared slightly larger than normal with depression (Figure 2). Optical coherence tomography (OCT; Heidelberg Spectralis, Germany) confirmed retinal detachment in the right eye (Figure 3), in contrast to the normal macular structure (Figure 4).

## 3 Discussion

### 3.1 Definition

MGS, also known as hereditary central optic disc glial cell abnormality syndrome, primarily manifests as abnormal visual acuity in infants and young children. Visual acuity is usually at the level of counting fingers, with a maximum of 0.05. Funduscopically, the optic disc and papilla are significantly enlarged, approximately 3–5 times the normal size, with a surrounding circular area of pigmentary change. The border between the optic disc and the peripapillary region is often blurred, and white glial tissue is present in the centre of the optic disc (2). Due to posterior displacement of the optic nerve head, retinal branches are frequently difficult to distinguish from the disc.

### 3.2 Pathogenesis

The pathogenesis of MGS remains incompletely understood. It is thought to involve incomplete closure of the upper end of the embryonic fissure, abnormal mesodermal differentiation, and defective formation of the optic nerve entrance. Abnormal development of glial cells in the central optic disc may also contribute (3, 4). Some propose that MGS, like optic nerve defects, results from abnormal foetal fissure closure, leading to a spectrum of clinical presentations ranging from optic disc depression to optic nerve coloboma (5). No clear pattern of genetic inheritance has been reported, and family history is typically absent (6). However, an association with a mutation in the paired box 6 (PAX6) gene PAX6 gene has been described (7). Currently, opinions on the pathogenesis of MGS remain diverse, and no consensus has been reached.

### 3.3 Pathology

Histopathology indicates that MGS is a congenital mesodermal lesion rather than a primary defect of the optic nerve or disc. Key features include axial posterior displacement of the optic nerve and funnel-shaped staphyloma surrounding the optic disc. Scleral ectasia is thought to result from developmental abnormalities of the posterior sclera. The optic nerve sheath may be affected and partially replaced by fibrous fat and smooth muscle tissue. Additionally, smooth muscle cells and adipocytes have been identified in the peripapillary sclera of staphyloma (8), and these smooth muscle cells may induce optic disc contraction (9). Manschot examined an enucleated eye from a child with MGS and found corneal Peters anomaly, angle dysplasia, congenital cataract, persistent hyperplastic primary vitreous, non-rhegmatogenous retinal detachment, retinal sickle folds, optic atrophy, absence of the lamina cribrosa, and peripheral scleral staphyloma. Dempster proposed that the clinical manifestations primarily relate to abnormal mesodermal and ectodermal growth, leading to defective posterior scleral closure and protrusion of the retina and optic nerve head (4).

### 3.4 Epidemiology

The reported prevalence of MGS is 2.6 per 100,000 individuals (3). The condition is more common in women, with a men-to-women ratio of approximately 1:2 (10). The vast majority of cases are monocular. Visual acuity in the affected eye has been poor since childhood, typically ranging from finger counting to 0.02. It is often accompanied by other congenital abnormalities, including strabismus, high myopia, and ocular tremor.

### 3.5 Diagnosis and differential diagnosis

The optic disc in MGS is funnel-shaped and symmetrically excavated, with the anterior optic nerve segment dilated and continuous with the vitreous cavity. Diagnostic modalities include routine fundus examination, fluorescein angiography, B-scan ultrasonography, computed tomography (CT), and magnetic resonance imaging (MRI). With these tools, the diagnosis is usually not difficult.

The fundus of MGS is characterized by a funnel-shaped enlarged optic disc depression, peripapillary choroidal retinal pigment ring, and central glial tissue coverage. B-scan ultrasonography reveals a posterior scleral defect at the posterior pole, with a funnel-shaped retrobulbar hypoechoic area connected to the vitreous cavity, occasionally containing irregular weak echoes (11). Colour Doppler ultrasonography can demonstrate a depression lacking normal optic nerve structure, with the optic nerve end adjacent to or at some distance from the depression floor. Perilesional blood flow signals are abundant, whereas signals within the depression are sparse or absent. The inverse “bottleneck” sign of the optic disc is a characteristic ultrasound finding (12). Fundus fluorescein angiography helps detect areas of peripheral capillary non-perfusion in MGS (13). Non-perfusion severity is generally classified into mild, moderate, severe, and extreme categories. Leakage occurs at the junction between the perfused and non-perfused retina. Fibrovascular proliferation and tractional retinal detachment have been observed in a few cases (14).

On CT, MGS typically shows significant optic nerve thickening, optic disc defects, funnel-shaped or cystic expansion of the optic nerve head, a low-density area continuous with the vitreous, increased density of the anterior optic nerve sheath, and continuity between the sheath and the scleral ring. The posterior sclera at the optic disc appears absent, with thinning and ectasia of the surrounding sclera. The crater-like optic disc depression is mainly confined to the disc itself, which is the primary CT feature of MGS. Additionally, cranial CT may reveal associated brain abnormalities such as encephalocele and corpus callosum dysplasia (15). The MRI manifestations of MGS were a funnel-shaped posterior wall of the optic disc with retinal elevation, distal optic nerve abnormalities, and subarachnoid disappearance, as well as scleral discontinuity (16).

MGS should be differentiated from peripapillary staphyloma and morning glory syndrome (presumably referring to other distinct entities). All three are rare optic disc anomalies. Peripapillary staphyloma lacks disc defects, glial proliferation, and abnormal vessels; its depression is deeper than in MGS and is typically unilateral. In what is here termed “chrysanthemum syndrome,” yellow-white membranous mounds are present on the optic disc, resembling a fully bloomed chrysanthemum. Although annular retinal choroidal pigment changes and vascular abnormalities are present, the optic disc itself is not depressed. Contrast-enhanced MRI shows mild enhancement, suggesting glial tissue on the disc surface (17).

### 3.6 Complications

MGS can be complicated by various conditions, with retinal detachment being the most common. Proposed mechanisms include abnormal contraction and traction of glial cells at the lesion site, leading to small clefts (18, 19). Vitreoretinal surgery may be performed if retinal detachment develops (20). Strabismus occurs in 90% of patients and may present as esotropia or exotropia (21, 22). Other associated ocular abnormalities include microphthalmos, congenital pupillary residual membrane, persistent hyperplastic primary vitreous, chronic simple glaucoma, complicated cataract, and non-rhegmatogenous retinal detachment (23, 24). Up to 45% of MGS cases may be associated with systemic abnormalities, including moyamoya disease, basal encephalocele, agenesis of the corpus callosum, and Posterior fossa brain malformations, large facial Haemangiomas, Arterial anomalies, Cardiac defects/coarctation of the aorta, and Eye anomalies (PHACE) syndrome (25). Nervous system abnormalities commonly include basal encephalocele, corpus callosal hypoplasia, moyamoya disease, and neurofibromatosis type II. Facial and skin manifestations include hypertelorism, flat nasal root, cleft lip, and cleft palate. Urinary system anomalies may include congenital renal defects, and endocrine abnormalities involve multiple hormonal axes, including pituitary–hypothalamic hormones.

### 3.7 Treatment

Early diagnosis and treatment of MGS are crucial. Domestic studies report that children with residual visual function may benefit from cycloplegic refraction, spectacle correction of ametropia, and standardized amblyopia training to improve visual acuity (25). The optimal age for amblyopia treatment is 2–8 years (26). For MGS complicated by retinal detachment, vitrectomy is an effective clinical option (27). Prophylactic near-optic nerve head laser photocoagulation has also been reported (28). However, additional long-term follow-up is required to validate its clinical utility and safety profile. Unfortunately, the patient in this report did not undergo a comprehensive systemic evaluation and, due to late diagnosis, did not receive follow-up treatment or monitoring. Notably, another study from the same year proposed an effective conservative treatment for serous retinal detachment associated with MGS. Multiple lines of evidence suggest that oral carbonic anhydrase inhibitors (e.g., acetazolamide) are effective primarily for serous retinal detachment without holes or clear tractional membranes. In contrast, vitrectomy remains the mainstay for cases with proliferative changes, clear traction, or rhegmatogenous detachment (29, 30).

## 4 Summary

MGS is a congenital optic disc dysplasia that leads to visual impairment. Its pathogenesis remains unknown, but the diagnosis is straightforward when characteristic clinical manifestations are combined with imaging findings. MGS may be accompanied by complications such as retinal detachment and strabismus. Early diagnosis and intervention are crucial for preserving or improving visual function in children. Treatment includes correction of strabismus and anisometropia, as well as prevention of amblyopia, primarily through spectacle correction and comprehensive amblyopia training. In cases complicated by retinal detachment, early vitreoretinal surgery is indicated. For patients with systemic abnormalities, multidisciplinary consultation involving ophthalmologists, neurologists, and physicians is recommended. Long-term follow-up is necessary to optimise patient outcomes.

## Data Availability

The original contributions presented in the study are included in the article/supplementary material, further inquiries can be directed to the corresponding author.
